# 

**Published:** 1846-10

**Authors:** 


### CONTENTS

OF NO. IV.

### ORIGINAL COMMUNICATIONS.

### ESSAYS AND CASES.

ART. I.—Thoughts on the Tongue as an Element in Diagnosis. By JOHN LAWSON, M.D., of Jamestown, Ohio. - 277

ART. II.—Remarks on Congestive Fever; with Cases. By FRANCIS H. GORDON, M.D., of Clinton College, Tennessee, - - - - - - - - - 299

ART. III.—Translation of a Chapter of the "Institutes of Medicine" of Heurnius, from the Latin. By Dr. JOHN G. AFFLECK, of Somerset, Ohio. - - - - 305

### REVIEWS.

ART. IV.—Chemistry, as Exemplifying the Wisdom and Beneficence of God. By GEORGE FOWNES, PH.D., Chemical Lecturer in the Middlesex-Hospital Medical School. New York: Wiley & Putnam. Philadelphia: J. W. Moore. 12 mo. pp. 158, 1844. - 313

### SELECTIONS FROM AMERICAN AND FOREIGN JOURNALS.

Characters of Broussais and Larrey. - - - - - - 341

Flogging in the British Army. - - - - - - 346

The Medical Profession—Quackery. - - - - - - 347

New Method of Preserving Organic Matters. - - - 349

Poisoning with Arsenic. - - - - - - - - 350

Treatment of Diabetes Mellitus by Balsam of Peru. - - 351

Death from the Effects of Cold Water Treatment. - - 351
Hooping Cough. - - - - - - - - - 352
Aconitum Napellus in Neuralgia. - - - - - 353
Billet-Doux to Baron Liebig. - - - - - - 356

ORIGINAL INTELLIGENCE.

Editorial Letter. - - - - - - - - - 357
Dr. Harris's Circular Letter, - - - - - - 364
Circular of the National Institute. - - - - - 366
Treatment of Leucorrhœa. - - - - - - 367
Electro-Magnetism in Intermittent Fever. - - - - 367
To the Subscribers of the Louisville Journal of Medicine and Surgery. - - - - - - - - - 368

